# Potential probiotic *Lactiplantibacillus plantarum* DS1800 extends lifespan and enhances stress resistance in *Caenorhabditis elegans* model

**DOI:** 10.3389/fphys.2024.1476096

**Published:** 2024-10-22

**Authors:** Seunghyun Kim, Yu-Ri Lee, Haneol Yang, Chan-Hyeok Park, Chan-Seok Yun, Byung-Chun Jang, Yeongjin Hong, Doo-Sang Park

**Affiliations:** ^1^ Korean Collection for Type Cultures, Biological Resource Center, Korea Research institute of Bioscience and Biotechnology, Jeongeup, Republic of Korea; ^2^ BioMedical Sciences Graduate Program (BMSGP), Chonnam National University Medical School, Gwangju, Republic of Korea; ^3^ KRIBB School of Bioscience, Korea University of Science and Technology, Daejeon, Republic of Korea

**Keywords:** *C. elegans*, *L. plantarum*, life extension, probiotics, stress resistance

## Abstract

Probiotics are live microorganisms that provide health benefits when administered in appropriate amounts by improving or restoring the balance of intestinal microbiota. Various functional probiotic products have been developed due to the growing interest in the health-promoting and anti-aging effects of enhancing the gut microbiome. *Lactiplantibacillus plantarum* species are known for their potential to extend lifespan. However, this activity is strain or isolation source specific, necessitating the identification of individual strain functionalities. This study used the *C. elegans* model to screen probiotics for life-extension effects and analyze their functions. The 43 lactic-acid bacteria strains isolated from fermented foods, breast milk, and human feces were subjected to longevity assays, and *L. plantarum* DS1800 was selected to demonstrate the most effective lifespan extension. The average lifespan of *Caenorhabditis elegans* fed DS1800 increased by 17.36% compared with those fed *Escherichia coli* OP50. Further analysis of the expression of key genes related to longevity revealed the high expression of the skinhead-1 (*skn-1*), antibacterial, and heat stress resistance genes via the p38 MAPK pathway. These expression patterns suggest that DS1800 extends the lifespan of *C. elegans* by enhancing its stress resistance and protecting it against pathogens. Additionally, DS1800 exhibited excellent intestinal adhesion, with 7.56% adhesion to HT-29 cells. Therefore, *L. plantarum* DS1800 is effective in extending the lifespan of *C. elegans* and can be used as a functional probiotic.

## 1 Introduction

Increasing interest in health and longevity has driven numerous investigations aimed at developing probiotics with relevant functionalities (Latif et al., 2023; Tsai et al., 2021; Zhang et al., 2022). Probiotics, defined by the World Health Organization and Food and Agriculture Organization, are live microorganisms that provide health benefits to hosts in appropriate amounts when administered (Hill et al., 2014). In particular, lactic acid bacteria (LAB) play a crucial role in enhancing human health and extending lifespans (Metchnikoff, 1908). Additionally, probiotics possess the capacity to inhibit pathogenic (Georgieva et al., 2015; Meleh et al., 2020), enhance intestinal barrier function (Judkins et al., 2020), and exert beneficial effects, such as immune modulation and neurotransmitter production, thereby modulating the gut microbiota and extending the host (Sánchez et al., 2017). Most species known for their probiotic properties belong to the *Lactobacillus* (Zheng et al., 2020) and *Bifidobacterium* genera, which were recently reclassified into 25 new genera. Notably, strains belonging to the *Lactiplantibacillus plantarum* species are recognized for their potential to extend lifespan due to their high intestinal adherence and ability to inhibit harmful bacteria (Pompa et al., 2023a). However, these functionalities are often strain specific or dependent on the isolation source rather than species specific (Tremonte et al., 2017), necessitating the identification of individual strain functionalities to select superior strains.

Influenced by Metchnikoff’s hypothesis on life extension, many studies have been conducted on prolonging lifespan (Riddle et al., 1997). Research related to this field has primarily focused on enhancing resistance to external stressors (Murakami and Johnson, 1996), inhibiting telomere attrition (Shalev et al., 2013; Yang et al., 1999), reducing the aging rate by lowering metabolic activity, and extending lifespan through caloric restriction (Nakagawa et al., 2012). Recently, several studies have used the *Caenorhabditis elegans* model, which has been optimized for longevity research, along with *in vitro* studies, to demonstrate its antimicrobial efficacy, increased resilience to external stressors, and lifespan extension via caloric restriction (Jeong et al., 2023; Kumar et al., 2020; Yun et al., 2022).

Since its initial application in neurobiology and genetics (Brenner, 1974) in the 1960s*, C. elegans* has been extensively used in various *in vivo* studies related to infection and immunity (Balla and Troemel, 2013; Cohen and Troemel, 2015). *C. elegans* offers several advantages, such as its small size, which facilitates culture in limited spaces, and its transparent body, which allows for internal observation. Additionally, its predominantly hermaphroditic nature enables rapid studies owing to its high fertility and short lifecycle. *C. elegans* is also widely used in studies related to lifespan regulation and aging inhibition because of the conservation of genes and signal transduction pathways that regulate lifespan (Kumar et al., 2020). Signaling pathways, such as the p38 mitogen-activated protein kinase (MAPK), insulin/insulin-like growth factor signaling (IIS), TGF-beta, and JNK pathways, are conserved in *C. elegans* (Roselli et al., 2019). Among these, the p38 MAPK pathway (MAPKKK-MAPKK-MAPK), also known as the NSY-1-SEK-1-PMK-1 pathway, is the oldest signaling pathway involved in nematode immunity and is essential for the activation of various immune responses. In mammals, the p38 MAPK pathway is crucial in cellular immune responses to inflammatory cytokines, such as interleukin-1 and tumor necrosis factor (Kim et al., 2002). In nematodes, the p38 MAPK pathway is involved in cellular processes, including stress response, detoxification, lipid metabolism, and immunity, by phosphorylating SKN-1, an ortholog of the human NF-E2-related factor (NRF-1) (Blackwell et al., 2015).

In this study, we used *C. elegans* to investigate the longevity-enhancing effects of various probiotic strains isolated from traditional fermented foods and human feces. We identified the L. plantarum DS1800 strain that exhibits exceptional longevity benefits and high intestinal adherence. Additionally, we confirmed the potential of developing new probiotics through antiaging and external stress-resistance experiments.

## 2 Materials and methods

### 2.1 Bacterial strains and culture conditions

**FIGURE 1:**
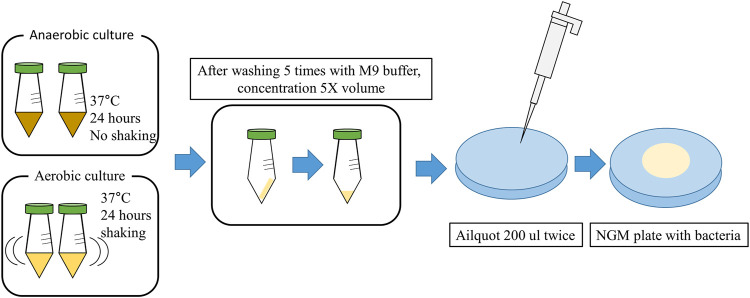
Graphic diagram showing the preparation of NGM agar plate with Bacteria (Details are provided in the Materials and Methods).

The 43 strains screened to identify lifespan extensions in *C. elegans* are listed in Supplementary Table S1. All the strains were obtained from the Bio R&D Product program (https://biorp.kribb.re.kr/) and Korean Collection for Type Cultures. These strains were cultured in De Man-Rogosa-Sharpe (MRS) broth at 37°C for 24–48 h under anaerobic conditions. As a control, *Escherichia coli* OP50 (OP50), a standard food for *C. elegans*, was obtained from the Korea Research Institute of Bioscience and Biotechnology (KRIBB) and used after 24 h of shaking incubation in LB broth at 37°C. *Lacticaseibacillus rhamnosus* GG KCTC 5033 (LGG), used as a positive control, was obtained from the Korea Research Institute of Bioscience and Biotechnology and incubated anaerobically in MRS broth at 37°C for 24 h. *Salmonella enterica* subsp*. enterica* KCTC 2015*,* obtained from the Korean Collection for Type Cultures, was incubated in Nutrient Broth at 37°C for 24 h with shaking and used as the pathogen. For this study, cells of each strain were adjusted to OD600, harvested using centrifugation (6,000 × g, 20 min), and washed seven times with an equal volume of M9 buffer. Finally, a five-fold concentrated bacterial suspension in M9 medium was prepared, and 400 μL of each suspension was dispensed onto Nematode Growth Medium (NGM) agar plates and dried at room temperature for 1 h. After drying, the plates were stored at 4°C for up to 2 weeks before being used for *C. elegans* culture and experiments. The NGM agar plate seeded with bacteria, as shown in Figure 1.

### 2.2 *Caenorhabditis elegans* culture conditions

The mutant strains of *C. elegans* used in this study, KU25 *pmk-1*(km25)IV and KU4 *sek-1*(km4)X, were obtained from the (KRIBB). The mutant strain, CF512 *fer-15*(b26)II; *fem-1*(hc17)IV, which is unable to produce offspring at 25°C, was provided by Seoul National University. All worms were fed a standard OP50 diet and maintained at 20°C prior to use in the study. For *C. elegans* egg preparation, adult *C. elegans* were cultured on NGM plates (60 mm diameter) supplemented with *E. coli* OP50 for 2 days at 20°C. After washing the densely populated plates three times with 1 mL of M9 buffer, eggs, and adults were collected and centrifuged at 3,000 rpm for 1 min to remove the supernatant. To separate the adults from the precipitate, 1 mL of bleach solution (100 μL of 5 N NaOH, 200 μL of 5% sodium hypochlorite, and 700 μL of Triple Distilled Water) was added to the suspension and centrifuged. This process was repeated two to three times to recover the eggs. The recovered eggs were washed three times with M9 buffer and incubated in M9 buffer at 20°C for 2 days. Hatched *C. elegans* were then transferred to NGM plates seeded with *E. coli* OP50 and incubated at 25°C until they reached the fourth larval stage (L4/young adults).

### 2.3 *Caenorhabditis elegans* life span assay

The *C. elegans* lifespan assay was conducted following a previously described method (Kim and Mylonakis, 2012) with some modifications. The longevity assay involved transferring young adult (L4) stage *C. elegans* (strain CF512) to NGM plates inoculated with the LAB strain used in the experiment, followed by incubation at 25°C for 20 days, with daily survival rate assessments. *Escherichia coli* OP50, a common bacterial diet for worms, was used as the control, and LGG served as the positive control. *C. elegans* were considered alive if they responded to a gentle touch with a platinum wire. Deaths caused by unusual factors, such as crawling away from the plate or sticking to the wall, were excluded from the analysis. The medium was refreshed every 3 days to ensure accuracy and prevent contamination, and approximately 150 worms were used in each experimental group.

### 2.4 RNA isolation and quatitative real-time polymerase chain reaction

Approximately 500 L4-stage fer-15; fem-1, sek-1, and pmk-1 mutants were collected in M9 buffer after 24 h of incubation at 25°C on plates spotted with OP50 or L. plantarum DS1800. RNA was isolated and purified using the RNeasy isolation kit (Qiagen, Hilden, Germany). The obtained RNA was quantified using a NanoDrop ND-1000 spectrophotometer (Thermo Scientific, Waltham, MA, United States), and 1 μg of RNA was used to synthesize cDNA with the iScript cDNA Synthesis Kit (Bio-Rad, Hercules, CA, United States). Primers for qRT-PCR were designed using NCBI Primer-BLAST (https://www.ncbi.nlm.nih.gov/tools/primer-blast/). The reference gene *act-1* was used for reliable gene expression analysis, and the primer sequences are listed in Supplementary Table S2 qRT-PCR was performed using a CFX Connect Real-Time PCR System (Bio-Rad) with SsoAdvanced Universal SYBR Green Supermix (Bio-Rad) in 96-well plates. The concentration of cDNA used was 20 ng, and the primer concentration was 10 pmol/μL. Relative gene expression levels were normalized using the 2^−ΔΔCT^ method.

### 2.5 Morphological characterization of *Caenorhabditis elegans*

Body size, body bending, and lipofuscin accumulation were measured to determine changes in the morphological characteristics of *C. elegans* (strain CF512). Body size was measured as previously described (Lee et al., 2015), with some modifications. L4 stage *C. elegans* were transferred to plates spotted with OP50, LGG, or DS1800 and cultured at 25°C for 4 days. Worm body size was observed every 24 h using a Nikon Eclipse TS100 microscope. ImageJ software (National Institutes of Health, Bethesda, MD, United States) was used to measure the body size of the worms. Twenty worms were used in each experiment. Body-bending measurements were performed according to a previously described method (Kumar et al., 2022) with some modifications. L4 stage *C. elegans* were transferred to plates spotted with OP50, LGG, or DS1800 and incubated at 25°C for 10 days. The worms were transferred to fresh OP50 plates for 30 min before measuring the body bending rate. The body bending rate per minute was measured using a Nikon Eclipse TS100 microscope with 10 worms for each assay. To analyze lipofuscin accumulation in the intestine, the method described by Pompa et al. (Pompa et al., 2023a) was applied with some modifications. L4 stage *C. elegans* were cultured on plates spotted with OP50, LGG, and DS1800 for 10 days at 25°C and then washed three times with M9 buffer. Worms were anesthetized by placing them on 5% agar pads coated with 10 mM sodium azide. Lipofuscin autofluorescence images were obtained using blue excitation light (405–488 nm), which is the channel of a Nikon Eclipse TS100 microscope. Fluorescence was quantified using the ImageJ software to measure lipofuscin levels. Ten worms per bacterial strain were used for each analysis.

### 2.6 Heat stress and oxidative stress assay

Thermotolerance and oxidative stress resistance assays were performed as previously described (Zhang et al., 2023), with minor modifications. For the thermotolerance assay, age-synchronized L4 stage *C. elegans* (strain CF512) were cultured in a 35°C incubator on plates individually spotted with OP50, LGG, or DS1800, and survival was measured every hour. For the oxidative stress resistance assay, L4 stage worms were cultured on NGM plates containing a final concentration of 0.03% H_2_O_2_ and spotted with OP50, LGG, and DS1800. The plates were incubated in a 25°C incubator, and survival was measured every hour until all worms were deceased. Survival was assessed as described previously for the *C. elegans* lifespan assay. Approximately 150 nematodes were used for each experimental group.

### 2.7 Assessment of pathogen infections

Age-synchronized nematodes (strain CF512) at the L4 stage were transferred to plates spotted with OP50, LGG, and DS1800 and incubated for 3 days in a 25°C incubator. They were then transferred to an NGM medium plated with the pathogen *S. enterica* subsp. enterica and incubated in a 25°C incubator. Nematode survival was determined every 24 h after transfer to plates containing the pathogen, with approximately 150 worms per experimental group (Sem and Rhen, 2012).

### 2.8 Bacterial attachment assay in *Caenorhabditis elegans* intestinal tract

A bacterial adhesion assay was performed as previously described (Yun et al., 2022). For the gut bacterial adhesion assay of *C. elegans* (strain CF512), plates seeded with OP50, LGG, and DS1800 were incubated for 24 h in a 25°C incubator. Subsequently, five worms from each plate were transferred to plates seeded with *S. enterica* subsp. *enterica. C. elegans* were collected on days 1, 3, and 5, and washed five times with M9 buffer. The washed worms were transferred to NGM plates and treated with 5 μL of gentamicin (25 μg/mL) for 5 min to remove bacteria attached to the body of *C. elegans*. The worms were washed five times with M9 buffer and disrupted in M9 buffer supplemented with 1% Triton X-100. Disrupted *C. elegans* were serially diluted in M9 buffer, cultured on MacConkey agar plates for 24 h, and colony-forming units (CFU) were measured.

### 2.9 Bacterial attachment assay in HT-29 cell

The cell adhesion assay was performed according to a previously described method (Khokhlova et al., 2023), with some modifications. The HT-29 human colorectal adenocarcinoma cell line was obtained from the Biological Resource Center of the Korea Biotechnology Institute and used to determine the intestinal cell adhesion ability of LGG, DS1800, and KCTC3108. HT-29 cells were grown and maintained in Dulbecco’s modified Eagle’s medium. For the adhesion assay, 12-well plates were inoculated with HT-29 cells at a concentration of 1.5 × 10^4^ cells/well and incubated until the cells reached 80% confluence. The cells were then treated with bacterial cultures (1 × 10^8^ CFU/mL) for 2 h. The bound bacteria were detached by treatment with 500 μL of 0.5% Triton X-100. The CFU of the adherent bacteria was calculated using serial dilution and plating on MRS agar plates. Bacterial adherence to cells was calculated using the following formula:

$$\text{Adherence}\;\left(\%\right)=\left(\text{CFU}\;\text{of}\;\text{adherent}\;\text{bacteria}\;/\;\text{CFU}\;\text{of}\;\text{initial}\;\text{inoculum}\right)\times 100$$

### 2.10 Statistical analyses

All the data were processed using Microsoft Excel (Microsoft Corporation, Redmond, WA, United States). *C. elegans* survival analysis was conducted using the Kaplan-Meier method (Bland and Altman, 1998), and differences were determined using the log-rank test (STATA6; STATA, College Station, TX, United States). Graphs were generated using SigmaPlot 14.5 (Inpixon, Palo Alto, CA, United States, RRID_003210). The results are form three independent replicates, with each experiment conducted once. The experimental results are presented as mean ± standard error of the mean. Significant differences between the two groups were determined using Student’s t-test.

## 3 Results

### 3.1 Screening of *L. plantarum* DS1800 through *Caenorhabditis elegans* lifespan analysis

**FIGURE 2:**
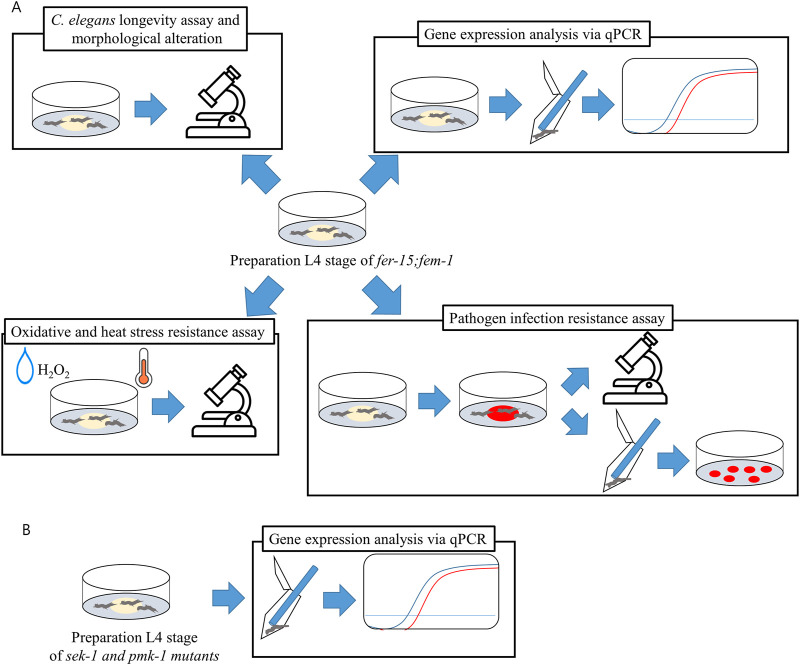
Schematic diagram of experimental design. *Caenorhabditis elegans* at the L4 stage were prepared on OP50, and all experiments were conducted with OP50, LGG, and DS1800. **(A)** Experimental design of CF512 *fer-15*(b26)II; *fem-1*(hc17)IV mutant. **(B)** Experimental design for gene expression in KU25 *pmk-1*(km25)IV and KU4 *sek-1*(km4)X mutants.

**FIGURE 3:**
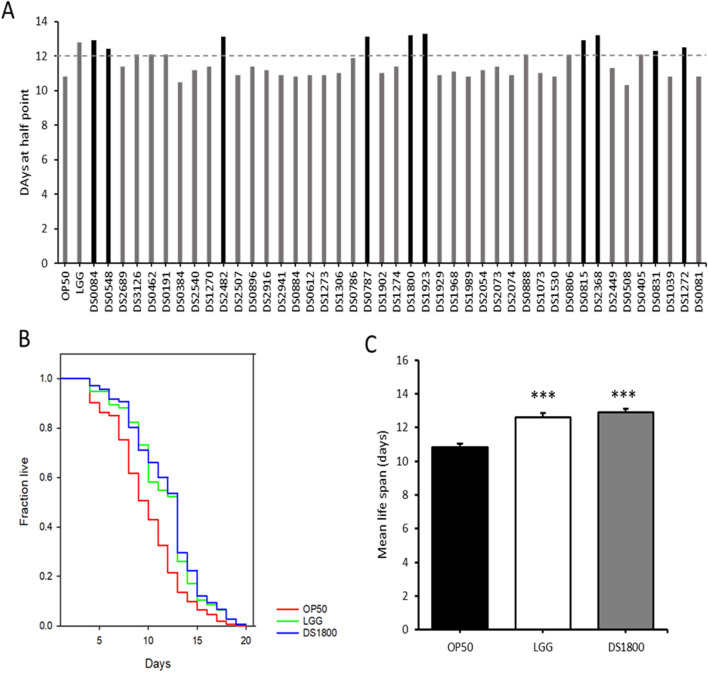
Screening of *Lactiplantibacillus plantarum* DS1800 through *Caenorhabditis elegans* lifespan analysis. **(A)** For the selection of lifespan-extending bacterial strains, the median survival days of worms fed each strain were documented. **(B, C)** Lifespan analysis and mean lifespan of worms fed with *Escherichia coli* OP50 (OP50), *Lacticaseibacillus rhamnosus* GG (LGG), and *L. plantarum* DS1800 (DS1800). (^***^ *p* < 0.0001).

In this study, LAB strains isolated from traditional fermented foods and human fece, those with significant lifespan-extending effects were selected, and the mechanisms underltying their lifespan extension were analyzed (Figures 2A, B). 43 LAB strains were fed to *C. elegans* for lifespan analysis. From these, ten strains exhibiting high median survival were initially selected (Figure 3A). The ten initially selected strains included *Lactobacillus acidophilus*, *Limosilactobacillus fermentum*, *Latilactobacillus sakei*, and five strains of *L. plantarum*, *Bifidobacterium bifidum*, and *Pediococcus pentosaceus*. All the strains showed a median survival of >12 days. Furthermore, the number of surviving *C. elegans* was monitored every 24 h to identify the superior strain, *L. plantarum* DS1800. *C. elegans* fed DS1800 exhibited a 17.4% increase in mean lifespan compared with *C. elegans* fed the standard diet of *E. coli* OP50. Additionally, *C. elegans* fed LGG showed a 15.2% increase in mean lifespan compared to those fed *E. coli* OP50 (Figures 3B, C).

### 3.2 Regulation of signaling gene expression in *Caenorhabditis elegans*

**FIGURE 4:**
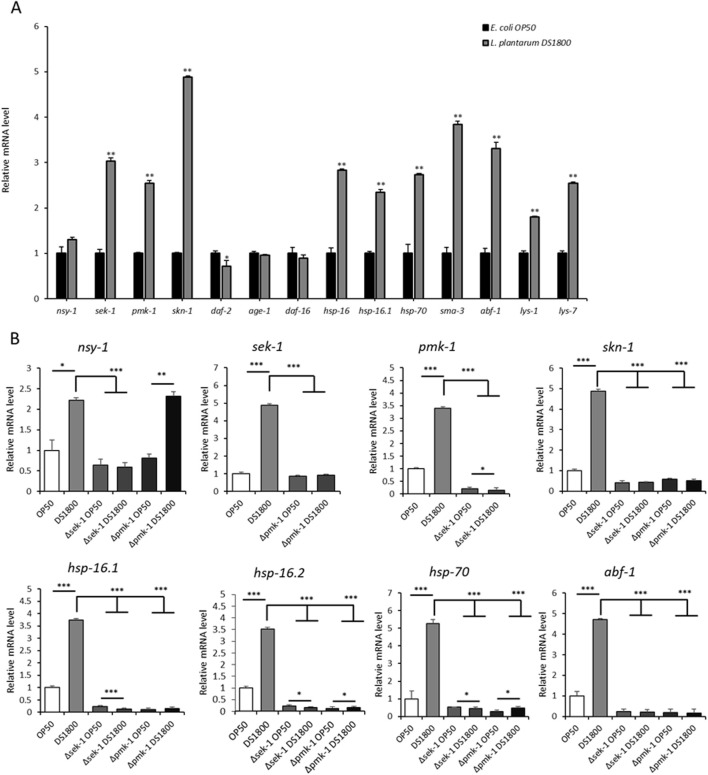
Expression changes of genes involved in immunity in *Caenorhabditis elegans.* **(A)** Gene expression changes in *fer-15;fem-1* mutant worms after 24-h consumption of OP50 and DS1800. **(B)** Gene expression changes in L4 stage *fer-15;fem-1*, *sek-1*, and *pmk-1* worms after 24-h consumption of OP50 and DS1800. (^*^ *p* < 0.05, ^**^ *p* < 0.001, ^***^ *p* < 0.0001).

To understand the molecular mechanisms underlying the lifespan-extension effects of DS1800 in *C. elegans*, we analyzed the expression of genes associated with longevity, stress response, and antimicrobial activity. The expression of genes related to PMK-1/p38 MAPK signaling (*sek-1, pmk-1,* and *skn-1*) showed a significant upregulation of 2.5-to 5-fold in worms fed DS1800 compared to those fed OP50, whereas the upstream gene, *nsy-1,* did not show a significant difference between the two groups. Additionally, genes related to heat shock proteins (*hsp-16, hsp-16.1,* and *hsp-70*) and antimicrobial peptides (*abf-1, lys-1,* and *lys-7*) were highly upregulated by 1.9- to 3.7-fold. No changes were observed in the expression of genes involved in the DAF-2/DAF-16 pathway (*daf-2, age-1,* and *daf-16*) (Figure 4A). We also examined changes in gene expression using *sek-1* and *pmk-1* loss-of-function mutant worms to understand the PMK-1/p38 MAPK signaling pathway and alterations in other genes. In Δ*sek-1* and Δ*pmk-1* worms, *sek-1* and *pmk-1* gene expression was not observed. Both mutant worms showed significantly lower expression levels of other genes, excluding *nsy-1*, than *fer-15;fem-1* worms (Figure 4B).

### 3.3 Effects of DS1800 under oxidative and heat stress conditions

**FIGURE 5:**
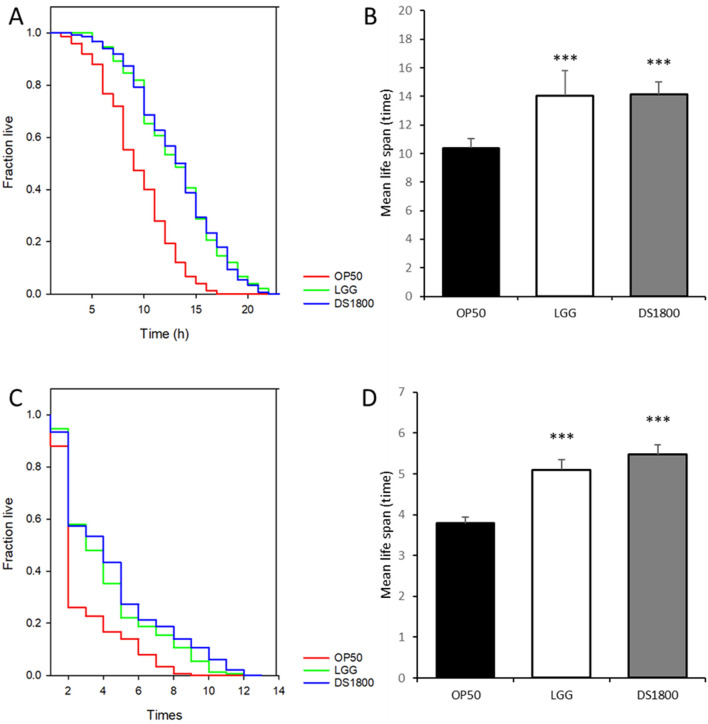
Effects of DS1800 under 35°C and H_2_O_2_ conditions. **(A, B)** Lifespan analysis and mean lifespan of worms fed with OP50, LGG, and DS1800 under 35°C conditions. **(C, D)** Lifespan analysis and mean lifespan of worms fed with OP50, LGG, and DS1800 under H_2_O_2_ conditions.

At 35°C, the mean lifespan of *C. elegans* fed with DS1800 increased by 30.69% compared to worms fed with OP50, while worms fed with LGG showed an increase of 30.08% (Figures 5A, B). Under oxidative stress conditions induced by H_2_O_2_ treatment, the mean lifespan of *C. elegans* fed DS1800 increased by 41.17% compared with that of worms fed OP50, and worms fed LGG showed an increase of 29.39% (Figures 5C, D).

### 3.4 Morphological alteration in *Caenorhabditis elegans* induced by DS1800

**FIGURE 6:**
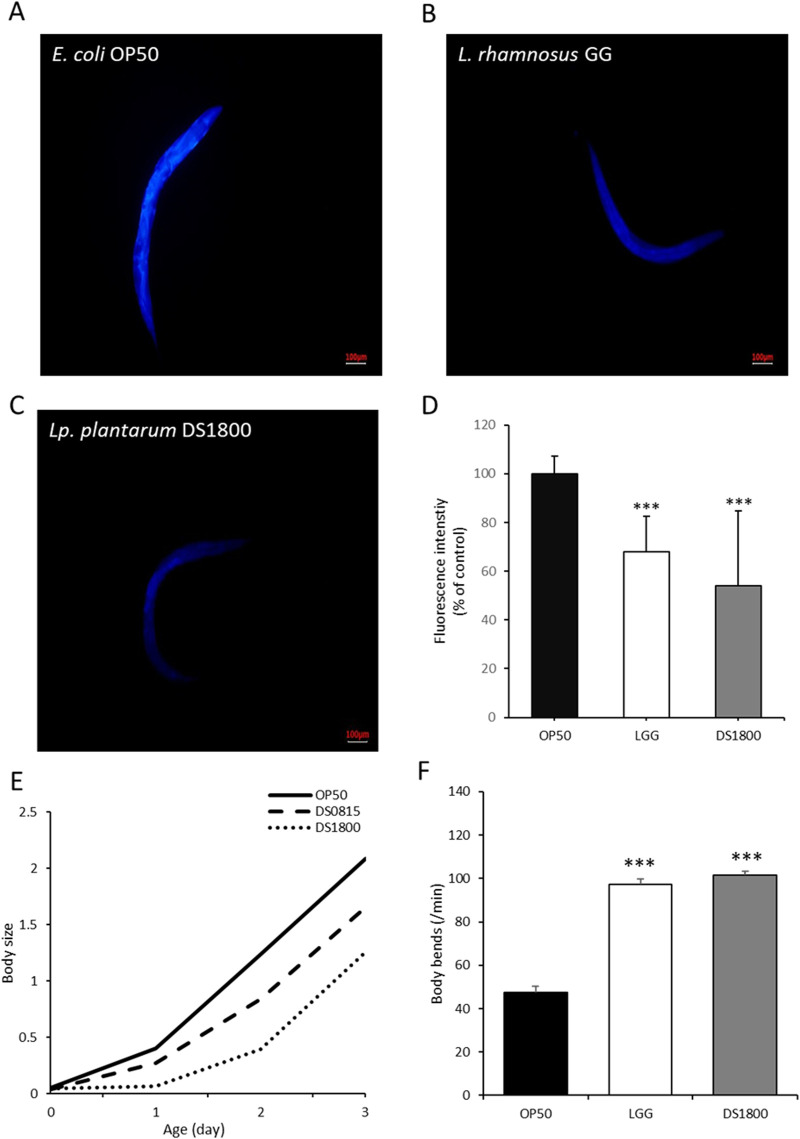
Morphological changes in *Caenorhabditis elegans* through the consumption of DS1800. Fluorescence microscopy images of lipofuscin in worms after 10 days of feeding with **(A)** OP50, **(B)** LGG, and **(C)** DS1800 for 10 days (^***^ *p* < 0.0001). **(D)** Relative fluorescence intensity of worms fed with OP50, LGG, and DS1800 for 10 days (% of OP50). **(E)** Body size changes of worms fed with OP50, LGG, and DS1800 from the L4 stage for 4 days. **(F)** Changes in body bending of worms following a 10-day diet of OP50, LGG, and DS1800.

Age-related morphological characteristics of *C. elegans*, including lipofuscin accumulation, body size, and body bending, were analyzed. Lipofuscin accumulation in worms fed DS1800 decreased by 46.12% compared to those fed OP50, whereas worms fed LGG showed a 32.04% reduction compared to those fed OP50 (Figures 6A–D). Body size changes were assessed by feeding L4 stage worms OP50, LGG, or DS1800 for 4 days. Worms fed DS1800 were significantly smaller than those fed OP50 (*p* < 0.001), with no significant difference in size compared to worms fed LGG (Figure 6E). The body-bending frequency of *C. elegans* was measured at 1-min intervals. Worms fed OP50 moved approximately 47 times, those fed DS1800 moved approximately 101 times, and those fed LGG moved approximately 97 times (Figure 6F).

### 3.5 Analysis of intestinal cell adhesion and pathogen infection resistance of *L. plantarum* DS1800

**FIGURE 7:**
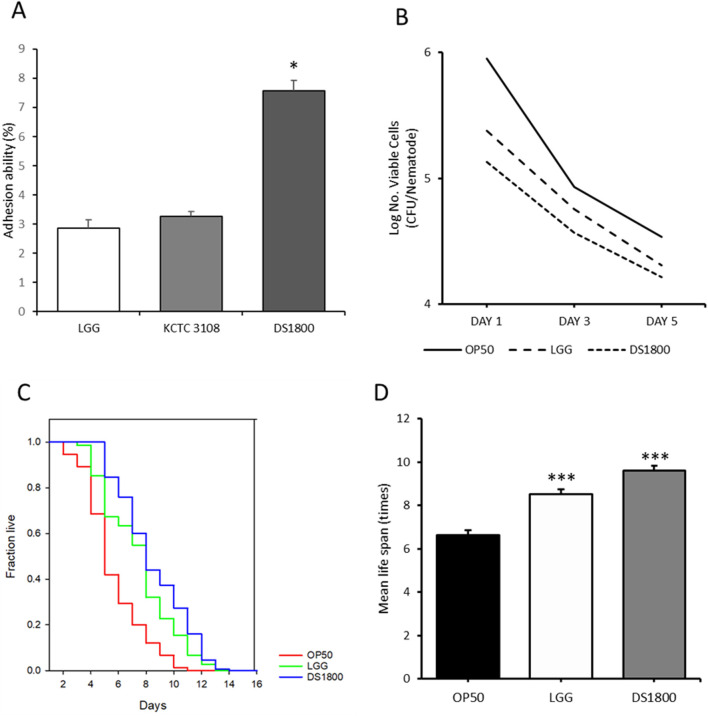
Potential intestinal protection of DS1800. **(A)** Intestinal adhesion ability of LGG, KCTC 3108, and DS1800 using HT-29 cells. (^***^ *p* < 0.001). **(B)** Bacterial counts of *Salmonella enterica* in the intestines of worms after 1, 3, and 5 days of feeding with *Salmonella enterica*, following a 24 h initial feeding with OP50, LGG, or DS1800. **(C, D)** Lifespan analysis and mean lifespan of worms fed with OP50, LGG, or DS1800 for 3 days and then exposed to *Salmonella enterica*.

To evaluate the protective potential of DS1800 in the intestine, cell adhesion ability was assessed using HT-29 cells. DS1800 exhibited an adhesion ability of 7.56%, which was significantly higher than those of *Lactiplantibacillus plantarum* KCTC3108^T^ (3.28%) and LGG (2.87%) (Figure 7A). To determine the resistance to pathogen infection in *C. elegans*, worms were exposed to the pathogenic strain *S. enterica* subsp. *enterica,* and subsequently analyzed for lifespan and intestinal pathogen adhesion. The mean lifespan of worms fed DS1800 and then exposed to the pathogen increased by 36.59% compared to that of worms fed OP50 and exposed to the pathogen. Additionally, the number of adherent pathogens decreased on days 1, 3, and 5. The mean lifespan of worms fed LGG and then exposed to the pathogen increased by 24.72%, and the number of adherent pathogens decreased on days 1, 3, and 5 (Figures 7B–D).

## 4 Discussion

Probiotics confer benefits to the host through immune modulation, metabolite release, or adhesion to host cells. LGG, used as a positive control in this study, is known for its role in maintaining gut homeostasis and protecting the intestinal barrier (Gao et al., 2019), and is one of the most extensively studied probiotics (Doron et al., 2005). Additionally, LGG extends the lifespan of *C. elegans* by regulating miRNA expression (Yun et al., 2022). By analyzing the lifespan extension effects of 43 LAB strains on *C. elegans*, we identified *L. plantarum* DS1800 as the most effective strain for lifespan extension. This result demonstrated a 17.4% increase in lifespan for L. plantarum DS1800 compared to OP50, which is significantly higher than the lifespan extension reported in other *L. plantarum* studies(Park et al., 2014; Heo et al., 2018). *Lactiplantibacillus plantarum* has been reported to exhibit antioxidant (Park et al., 2011) and antimicrobial activities (Hashemi et al., 2017), and several strains are known for their lifespan extension properties (Kumar et al., 2022; Pompa et al., 2023b). Furthermore, *L. plantarum* is widely used as a probiotic because of its high biomass productivity and stability. The newly isolated *L. plantarum* DS1800 demonstrates great potential for application as a probiotic.

The innate immune pathways in *C. elegans* include the IIS and p38 MAPK pathways, which are conserved in both humans and nematodes (Roselli et al., 2019). The IIS pathway is initiated by the activation of dauer formation-2 (DAF-2) and inhibits the activity of the Forkhead FoxO transcription factor DAF-16, which is associated with lifespan extension (Fontana et al., 2010). The p38 MAPK pathway is the oldest conserved innate immune pathway in nematodes and involves the NSY-1-SEK-1-PMK-1 cascade to activate SKN-1 (Kim et al., 2002). SKN-1 promotes the expression of antioxidant and detoxification enzymes, thereby activating genes involved in the stress response (Blackwell et al., 2015). We analyzed gene expression to elucidate the impact of DS1800 consumption on innate immune signaling pathways in *C. elegans*. Compared to worms fed OP50, those fed DS1800 showed significant downregulation of the IIS pathway-related gene *daf-2*, whereas no significant changes were observed in the expression of *age-1* and *daf-16*. The expression of genes associated with the p38 MAPK pathway (*sek-1*, *pmk-1*, and *skn-1*) was significantly upregulated, with fold changes of 2.9, 2.5, and 4.7, respectively. These results demonstrated significantly higher expression levels compared to those observed with other *L. plantarum* studies (Pompa et al., 2023b; Kumar et al., 2022). Additionally, genes related to longevity, such as heat shock proteins (*hsp-16*, *hsp-16.1*, and *hsp-70*) and antimicrobial peptides (*abf-1*, *lys-1*, and *lys-7*), were upregulated by approximately 1.9- to 3.7-fold. To further investigate the role of the p38 MAPK pathway in the expression of heat shock and antimicrobial genes, we analyzed the gene expression in *sek-1* and *pmk-1* loss-of-function mutant worms. In *sek-1* mutants, *sek-1* expression was absent, and the downstream genes *pmk-1* and *skn-1* exhibited significantly reduced expression levels. The upstream gene *nsy-1* did not show a significant difference in expression between worms fed OP50 and those fed DS1800. In *pmk-1* mutants, *pmk-1* expression was absent, and *skn-1* expression was reduced. Similar to *sek-1* mutants, no significant difference existed in the expression of the upstream genes. Both mutant strains exhibited a drastic decrease in the expression of *hsp-16.1*, *hsp-16.2*, *hsp-70*, and *abf-1*. These results suggest that DS1800 modulates the expression of heat shock and antimicrobial genes via the p38 MAPK pathway in *C. elegans*. Collectively, our findings indicate that DS1800 extends lifespan, enhances oxidative and heat stress resistance, and exerts antimicrobial effects in *C. elegans*, primarily through the p38 MAPK pathway, independent of the IIS pathway.

Heat shock and oxidative stress accelerate cellular aging by disrupting protein homeostasis, and cells possess pathways for detecting and repairing stress-induced damage (Ben-Zvi et al., 2009). Based on the observed upregulation of heat shock proteins and oxidative stress resistance genes in this study, we confirmed that DS1800 could enhance stress resistance in worms. To assess whether DS1800 improves heat shock and oxidative stress resistance in worms, we cultured them at 35°C. The mean lifespan of *C. elegans* fed with DS1800 increased by 30.6% under 35°C conditions and 36.3% under H_2_O_2_ conditions compared to worms fed with OP50. These findings suggest that DS1800 enhances resistance to environmental stressors, such as heat and oxidative stress, thereby extending the overall biological lifespan.

Longevity in *C. elegans* is associated with biomarkers such as body size, body bending, and lipofuscin accumulation (Son et al., 2019). Reduced body size can preserve cellular proliferation capacity and extend lifespan (McCulloch and Gems, 2003), whereas changes in locomotion, such as body bending, are closely related to longevity (Hosono et al., 1980). Lipofuscin is a fluorescent substance that accumulates in post-mitotic cells and serves as an indicator of aging in animals such as *C. elegans* (Terman and Brunk, 2006). These biomarkers are critical indicators for assessing age-related changes and are essential for understanding the potential health benefits of probiotics. We found that worms fed DS1800 exhibited smaller body size, reduced body bending, and lower lipofuscin accumulation than those fed OP50. These findings suggest that DS1800 supplementation delays aging in *C. elegans*.

One of the mechanisms through which probiotics contribute to maintaining human health and preventing diseases is their antimicrobial activity. The antimicrobial action of probiotics refers to their ability to inhibit or eliminate the growth of other microorganisms through various mechanisms (Cotter et al., 2005; Hernández-González et al., 2021). Probiotics exert their antimicrobial effects in the body by 1) lowering the pH of the surrounding environment through the production of organic acids, thereby inhibiting the growth of pathogenic bacteria (Tejero-Sariñena et al., 2012); 2) maintaining gut microbial balance and preventing the overgrowth of harmful microorganisms through the production of antimicrobial peptides, such as bacteriocins (Hernández-González et al., 2021); 3) directly inhibiting pathogenic microorganisms by competing for adhesion sites in the gut (Bernet et al., 1994); and 4) enhancing the host’s immune defense mechanisms against pathogenic microorganisms. In this study, we analyzed the survival rate of *C. elegans* following exposure to the intestinal pathogen *S. enterica*. Worms fed DS1800 showed a 36.59% increase in the mean lifespan compared to those fed OP50 upon pathogen exposure. This increase was greater than the 24.68% increase observed in worms fed LGG, which was used as the positive control. These results indicated that DS1800 significantly enhanced the resistance to stress induced by pathogen exposure. Furthermore, DS1800 exhibited a 7.56% adhesion rate to HT-29 cells in the intestinal bacterial adhesion assay. This rate was 2.3–2.6 times higher than the adhesion rates of 3.28% for the standard strain *L. plantarum* KCTC3108% and 2.87% for LGG. This superior adhesion capability is crucial for maintaining beneficial effects in the gut, including survival, niche occupation, and inhibition of harmful bacteria. Therefore, the *L. plantarum* DS1800, which demonstrated excellent lifespan extension effects in *C. elegans*, could be a promising probiotic candidate. It enhances resistance to oxidative and heat stress, inhibits pathogen proliferation in the gut, and supports gut health.

In conclusion, we identified *L. plantarum* DS1800, a strain with significant lifespan-extending effects, from 43 strains isolated from traditional fermented foods and human feces. We found that DS1800 enhances the MAPK signaling pathway in *C. elegans*, leading to increased resistance to oxidative and heat stress, as well as protection against pathogenic infections. However, further validation using *in vivo* studies in more complex animal or human systems is required to confirm its potential health benefits for humans.

## Data Availability

The datasets presented in this study can be found in online repositories. The names of the repository/repositories and accession number(s) can be found in the article/Supplementary Material.
