# Supplementary material for: Potential probiotic Lactiplantibacillus plantarum DS1800 extends lifespan and enhances stress resistance in Caenorhabditis elegans model
Source: Front Physiol. 2024 Oct 22;15:1476096. doi: 10.3389/fphys.2024.1476096 (PMC11534597; doi:10.3389/fphys.2024.1476096)
Supplement: Supplementary file 1 [file DataSheet1.PDF]

Supplementary Material

Potential Probiotic Lactiplantibacillus plantarum DS1800 Extends Lifespan and Enhances Stress Resistance in Caenorhabditis elegans Model

Seunghyun Kim[1, 2], Yu-Ri Lee[1], Haneol Yang[1], Chan-Hyeok Park[1], Chan-Seok Yun[1], Byung-Chun Jang[1, 2], Yeongjin Hong[2*], Doo-Sang Park[1, 3*]

[1]Korean Collection for Type Cultures, Biological Resource Center, Korea Research institute of Bioscience and Biotechnology, Jeongeup, 56212, Republic of Korea

[2] BioMedical Sciences Graduate Program (BMSGP), Chonnam National University Medical School, Hwasun, 58128, Republic of Korea

[3]KRIBB School of Bioscience, Korea University of Science and Technology, Daejeon, 34113, Republic of Korea

Running title: Life extension effect of *L. plantarum* in *C. elegans*

*** Correspondence:**
Doo-Sang Park
Tel: +82-63-570-5640, E-mail: dspark@kribb.re.kr

Yeongjin Hong
Tel: +82-61-379-8478, E-mail: yjhong@jnu.ac.kr

Supplementary Tables

Supplementary Table 1. List of 43 strains screened for lifespan extension

Species	Strain No.	Origins	Deposit No.
Lactobacillus acidophilus	DS0084	Yogurt	Isolated
Limosilactobacillus fermentum	DS0548	Infant feces	BP1883262
Limosilactobacillus fermentum	DS2689	Infant feces	BP1884565
Limosilactobacillus fermentum	DS3126	Infant feces	BP1884980
Limosilactobacillus gasseri	DS0462	Breast milk	BP1883163
Limosilactobacillus reuteri	DS0191	Pig small intestine	KCTC14231BP
Limosilactobacillus reuteri	DS0384	Infant feces	KCTC14164BP
Limosilactobacillus reuteri	DS2540	Chicken feces	BP1884422
Latilactobacillus sakei subsp. *sakei*	DS1270	Kimchi	BP1421263
Latilactobacillus sakei	DS2482	Kimchi	BP1882521
Latilactobacillus sakei	DS2507	Kimchi	BP1882546
Lacticaseibacillus paracasei	DS0896	Infant feces	BP1883409
Lacticaseibacillus paracasei subsp. *tolerans*	DS2916	Infant feces	BP1884786
Lacticaseibacillus paracasei	DS2941	Infant feces	BP1884811
Lactiplantibacillus pentosus	DS0884	Infant feces	BP1883399
Lactiplantibacillus pentosus	DS0612	Kimchi	BP1883339
Lactiplantibacillus pentosus	DS1273	Kimchi	BP1421266
Lactiplantibacillus pentosus	DS1306	Kimchi	BP1421295
Lactiplantibacillus plantarum	DS0786	Kimchi	BP1883390
Lactiplantibacillus plantarum	DS0787	Kimchi	BP1883391
Lactiplantibacillus plantarum	DS1902	Kimchi	BP1883956
Lactiplantibacillus plantarum	DS1274	Kimchi	BP1883526
Lactiplantibacillus plantarum	DS1800	Kimchi	BP1883740
Lactiplantibacillus plantarum	DS1923	Kimchi	BP1883977
Lactiplantibacillus plantarum	DS1929	Kimchi	BP1883983
Lactiplantibacillus plantarum	DS1968	Kimchi	BP1884021

Lactiplantibacillus plantarum	DS1989	Kimchi	BP1884041
Lactiplantibacillus plantarum	DS2054	Kimchi	BP1884106
Lactiplantibacillus plantarum	DS2073	Kimchi	BP1884125
Lactiplantibacillus plantarum	DS2074	Kimchi	BP1884126
Lactiplantibacillus plantarum	DS0888	Infant feces	BP1883383
Lactiplantibacillus plantarum	DS1073	Infant feces	BP1883494
Lactiplantibacillus plantarum	DS1530	Adult feces	BP1883589
Lactiplantibacillus plantarum	DS0806	Fermented pork	BP1883374
Lactiplantibacillus plantarum	DS0815	Fermented pork	BP1883382
Lactiplantibacillus plantarum	DS2368	Gallstone	BP1884367
Lactiplantibacillus plantarum	DS2449	Pickle	BP1884415
Lacticaseibacillus rhamnosus	DS0508	Infant feces	KCTC13504BP
Bifidobacterium animalis	DS0405	Infant feces	BP1883454
Bifidobacterium bifidum	DS0831	Infant feces	BP1883274
Bifidobacterium longum subsp. *longum*	DS1039	Infant feces	BP1883435
Pediococcus pentosaceus	DS1272	Kimchi	BP1421265
Streptococcus salivarius subsp. *thermolhilus*	DS0081	Yogurt	Isolated

Supplementary Table 2. Primer sets used for quantitative RT-PCR

Gene Name	Primer	Sequence
nsy-1	forward	5'-AGTTGTAGACACGCCGGTTA-3'
	reverse	5'-TGCACTGTTCAGGCTTTCAC-3'
sek-1	forward	5'-GCCGATGGAAAGTGGTTTTA-3'
	reverse	5'-TAAACGGCATCGCCAATAAT-3'
pmk-1	forward	5'-ACTTCATCCGACTCCACGAG-3'
	reverse	5'-CAGCAGCACAAACAGTTCCA-3'
skn-1	forward	5'-GTGTCGGCGTTCCAGATTT-3'
	reverse	5'-CAATAATGATGCTTACCGCGT-3'
daf-2	forward	5'-GCCCGAATGTTGTGAAAACT-3'
	reverse	5'-CCAGTGCTTCTGAATCGTCA-3'
age-1	forward	5'-TTCGTAGAGTGGTTTTGGGC-3'
	reverse	5'-GGTTTCTTCAATTGCTGGA-3'
daf-16	forward	5'-TCCTCATTCACTCCCGATTC-3'
	reverse	5'-CCGGTGTATTCATGAACGTG-3'
hsp-16	forward	5'-CTATGGGCCCAAAAGGAAGAAACGTG-3'
	reverse	5'-GGATTTCGCGACGGTGACTCCGTCC-3'
hsp-16.1	forward	5'-GCAGAGGCTCTCCATCTGAA-3'
	reverse	5'-GCTTGAACTGCGAGACATTG-3'
hsp-16.2	forward	5'-CTATTTCCGTCCAGCTCAAC-3'
	reverse	5'-TTTGTTCAACGGGGCGCTTGC-3'
hsp-70	forward	5'-ACGGGATGCTGTCATTACTG-3'
	reverse	5'-ATGTACCTCCTCCCAAATCG-3'
sma-3	forward	5'-CCACCGCACAGTCAATTATG-3'
	reverse	5'-ATCTTCTCCCCAGCCCTTTA-3'
abf-1	forward	5'-GTACAGCACAGAAATGCATGACCGG-3'
	reverse	5'-GGCGTTTGAACAACCTCCACAGAAGC-3'
lys-1	forward	5'-GGATTCAGGTTACCTCCCCAGCC-3'
	reverse	5'-GGTGTAGATTCCGACAGTCAGTCCG-3'
lys-7	forward	5'-GCGGGTTATTGTGCAGTTTT-3'

	reverse	5'-TCAATTCCGAGTCCAGCTTT-3'
act-1	forward	5'-GCTGGACGTGATCTTACTGATTACC-3'
	reverse	5'-GTAGCAGAGCTTCTCCTTGATGTC-3'
